# Comparison of mortality prediction models for road traffic accidents: an ensemble technique for imbalanced data

**DOI:** 10.1186/s12889-022-13719-3

**Published:** 2022-08-02

**Authors:** Yookyung Boo, Youngjin Choi

**Affiliations:** 1 Department of Health Administration, Dankook University, Cheonan, 31116 South Korea; 2 Department of Healthcare Management, Eulji University, Seongnam, 13135 South Korea

**Keywords:** Imbalanced data, Ensemble method, Road traffic accident injury, Mortality prediction, Machine learning

## Abstract

**Background:**

Injuries caused by RTA are classified under the International Classification of Diseases-10 as ‘S00-T99’ and represent imbalanced samples with a mortality rate of only 1.2% among all RTA victims. To predict the characteristics of external causes of road traffic accident (RTA) injuries and mortality, we compared performances based on differences in the correction and classification techniques for imbalanced samples.

**Methods:**

The present study extracted and utilized data spanning over a 5-year period (2013–2017) from the Korean National Hospital Discharge In-depth Injury Survey (KNHDS), a national level survey conducted by the Korea Disease Control and Prevention Agency, A total of eight variables were used in the prediction, including patient, accident, and injury/disease characteristics. As the data was imbalanced, a sample consisting of only severe injuries was constructed and compared against the total sample. Considering the characteristics of the samples, preprocessing was performed in the study. The samples were standardized first, considering that they contained many variables with different units. Among the ensemble techniques for classification, the present study utilized Random Forest, Extra-Trees, and XGBoost. Four different over- and under-sampling techniques were used to compare the performance of algorithms using “accuracy”, “precision”, “recall”, “F1”, and “MCC”.

**Results:**

The results showed that among the prediction techniques, XGBoost had the best performance. While the synthetic minority oversampling technique (SMOTE), a type of over-sampling, also demonstrated a certain level of performance, under-sampling was the most superior. Overall, prediction by the XGBoost model with samples using SMOTE produced the best results.

**Conclusion:**

This study presented the results of an empirical comparison of the validity of sampling techniques and classification algorithms that affect the accuracy of imbalanced samples by combining two techniques. The findings could be used as reference data in classification analyses of imbalanced data in the medical field.

## Background

Road traffic accidents (RTAs) mortality is affected by the circumstances of the accident, including the type of vehicle, the number of passengers, their personal characteristics, and accident-induced injury/disease factors. Among RTAs, “vehicle-on-vehicle collisions” account for 73.0% of all RTAs, while the parts of the body that are most often injured are the “head”, “chest”, and “face” in that order [1]. With respect to the types of RTA injuries, “sprains and dislocations” are the most common type, followed by “fractures”, “superficial injury” and “internal organ damage.” The average length of hospital stay due to RTA injury is approximately two weeks, but patients often experience sequelae and disability due to the accident. However, RTA mortality rate is low, accounting for only 2–3% of all RTA patients [2]. RTA injuries often cause more serious dysfunction compared to other forms of blunt trauma, which has the potential to cause a significant social burden [3]. Despite the low RTA mortality rate, RTA injuries require a national management system and there is an urgent need to predict RTA mortality.

In road traffic, there are many studies focusing on accident environment factors such as road conditions and climate, but there are not many medical approaches due to injuries. However, the direct cause of mortality is injury, and complex injuries such as internal organ injury and amputation and crush are known to be severe injuries that lead to mortality. In particular, if the head, neck, and abdomen are the primary site of injury, age, and surgery are performed, the probability of mortality increases [4].

In medicine, ML algorithms are being used to predict the mortality risk of diseases. Prediction of in-hospital mortality for heart and coronary disease, cancer, patients at emergency departments, and after cardiac surgery have many applications, and these studies use clinical features such as vital signs and Glasgow coma scale as predictors [5, 6].

Classifications based on logistic regression models and decision-based techniques are used to predict mortality [7–9]. Recently, there has been an increasing interest in the ensemble technique for improving the performance of classifications. In particular, the performance of decision tree continues to improve, and consequently, upgraded models for Random Forest, Extra-Trees, and XGBoost have been introduced. Improvements have been made with the application of bagging and boosting techniques [10–12]. When the classes are divided into “survivors” and “deceased”, there is a large disparity in the number of observations per class, and this is referred to as imbalanced data. If such imbalanced data is used for classification, data from the class with the higher number of observations have a dominant role in generating the classifier [13, 14]. However, information contained in the class with less number of observations is also important; this presents a difficulty in classification modelling [15].

There are two main approaches to random resampling for imbalanced classification; they are over-sampling and under-sampling. Over-sampling methods can be divided into simple random over-sampling, synthetic minority oversampling technique (SMOTE), and cost-weighting that assigns weight to samples in consideration of their distribution [16]. While there are studies that have reported that under-sampling causes decline in performance due to specific data included in the sample being deleted, other studies have reported that under-sampling can produce superior performance than over-sampling in some cases due to distortion that may occur during the over-sampling process [17, 18]. The present study compared the algorithms that applied the ensemble technique with the conventional method for predicting RTA mortality.

## Methods

### Study samples

The study used data from the Korean National Hospital Discharge In-depth Injury Survey (KNHDS) conducted by the Korea Disease Control and Prevention Agency, covering a 5-year period between 2013 and 2017 to determine mortality related to external injuries caused by road traffic accidents. The survey population in KNHDS was defined as all patients who were discharged from general hospitals having 100 or more beds. KNHDS data items consist of the type of medical institution, patient demographics, geographic area, dates of admission, type of disease, and treatment information. Moreover, in depth information regarding the injury and the code of the external cause for injury were additionally investigated in injured patients who were discharged. Primary diagnosis was based on the International Classification of Diseases, 10th edition (ICD-10) from the World Health Organization (WHO) and Korean Classification of Disease, 7th edition (KCD-7).

**Fig. 1:**
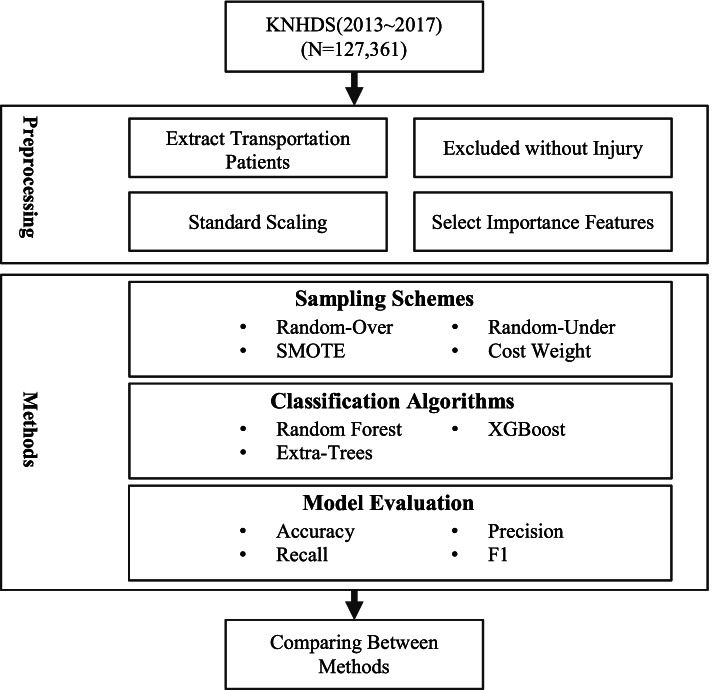
Study workflow

The present study extracted RTA data from the 2013–2017 data set of KNHDS. Variables extracted by importance analysis were standardized. Data sets obtained after pre-processing were assessed for sampling schemes and classification algorithms using the model assessment method (Fig. 1).

### Definition of variables

The present study used RTA mortality as the classification criterion. Mortality was classified as “survived” or “died” using the treatment outcome among the survey items. Moreover, data regarding patient demographics, accident circumstances, and injury and disease characteristics were used to determine the factors that influence mortality and the classification criterion [19, 20]. For patient demographics, age was selected by considering existing studies on increased risk of RTA with age. Accident characteristics included type of accident and role of the injured person in the accident. Injured person’s role in accident was further classified into five attributes including “driver,” “passenger” and “pedestrian.” Injury and disease characteristics included “primary diagnosis”, “patterns of principal injury,” “site of injury,” “operation status” and “type of injury” [21]. The site of injury was further classified as “head and neck,” “spine and back,” “trunk,” “upper extremities,” “lower extremities” and “others” according to the classification codes in the guidelines for usage of KNHDS raw data. Pertaining to patterns of injuries, “superficial injury,” “open wound,” “sprain” and “dislocation” were classified into mild injuries, while “other injuries” were defined as severe injuries. Type of injury was further classified as “single-site” and “multiple-site injury,” while the operation status was defined as “yes” if a date for primary operation appeared in the record [21, 22]. KNHDS data were analyzed using Python 3.8.0 (Python Software Foundation, Delaware, USA) after data cleansing using Excel (Microsoft Excel 2016, Microsoft Corp., Redmond, WA, USA).

### Analytical techniques

The present study applied sampling techniques for the correction of imbalanced data. Classification models are used as scales for assessing the prediction accuracy, but because they are created based on balanced data, they are inappropriate for imbalanced data [14]. Imbalanced classification is specifically difficult because of the severely skewed class distribution and the unequal misclassification costs.

Excessive distribution of the majority class may lead to encroachment of the boundary with the minority class, and as a result, the minority class generally overlaps a part of the majority class space [23].

The difficulty of imbalanced classification is compounded by properties such as dataset size, label noise, and data distribution. Most of the predictions will correspond to the majority class and treat the minority class features as noise in the data. Due to over-sampling the proportion of the minority class may increase and due to under-sampling the proportion of the majority class may reduce [24]. To address this problem, various re-sampling techniques including over-sampling, under-sampling, and SMOTE have been used [25].

The study used the ensemble technique for predicting binary classification. Random Forest is a classification model that combines bagging and decision tree model. It is a model that aggregates multitude of decision trees outputted to determine the final prediction values according to the average or majority voting [26].

Boosting model is an ensemble technique developed by Schapire [27], which was created to learn decision trees sequentially, each trying to improve on the errors of its predecessor. XGBoost is a gradient boosting method recently developed by Chen [28], which proved its worth in various machine-learning competitions. Owing to system optimization through parallelization and pruning; performance enhancement through regularization term and weighted quantile algorithm, it is faster than conventional gradient boosting machines and allows a generalized model to be obtained. Moreover, it also offers the advantage of being able to use graphic processor units due to parallelization. During the training process, XGBoost is trained to minimize the objective function consisting of loss function and regularization term. The regularization term is a term that has been added to limit model overfitting. Prediction value and objective function are as shown below.

$$\mathrm{y}\hat{\phantom{a}}\mathrm{i}={\textstyle\sum_{k=1}^K}\ f_k\left(x_i\right)$$

$$\mathrm{Obj}=\sum\nolimits_{i=1}^nl\left(\mathrm{y}\hat{\phantom{a}}\mathrm{i},\mathrm{yi}\right)+\sum\nolimits_{k=1}^K\varOmega \left({f}_k\right)$$

The Extra-Trees algorithm, another boosting model, is an ensemble learning technique that cumulatively summarizes decision tree outputs. The Extra-Trees algorithm is different from other tree-based ensemble techniques as it divides the node by selecting a random cut point and uses the entire learning sample for growing the tree [29]. And, we used 5-fold cross validation, 4 fold are utilized for the development of models and the rest one hold is utilized for the validation of models performance.

## Results

### Sample characteristics

The study sample included a total of 55,279 participants with a higher percentage of males (*n* = 32,936, 59.6%) than females (*n* = 22,343, 40.4%). The role of the injured person at the time of the accident was as follows: “driver” (40.4%), “pedestrian” (17.0%), “passenger” (16.0%). The primary site of injuries were the “abdomen and back” (20.1%), “head” (20.0%), and “neck” (19.0%), which when combined accounted for 60% of all injuries. With respect to the pattern of principal injury, the most common was “sprain and dislocation” (43.5%), followed by “fracture” (20.0%) and “superficial injury” (16.3%). Of the 55,279 inpatients injured, only 670 (1.2%) died in the hospital. Moreover, the average age of the patients with mild injury was 62.28 years, which was in stark contrast to the average age of 42.79 years for the complete sample. The general characteristics of the study population are presented in Table 1.

**Table 1:** General characteristics of the study population

Items	Frequency (%)
**Sex**	
Male	32,936 (59.6)
Female	22,343 (40.4)
**Severity of injury - Mild**	
Superficial injury	9037 (16.3)
Open wound	1825 (3.3)
Sprain/dislocation	24,049 (43.5)
**Severity of injury - Severe**	
Fracture	11,035 (20.0)
Nerve injury	289 (0.5)
Blood vessel injury	87 (0.2)
Internal organ injury	8147 (14.7)
Muscle injury	457 (0.8)
Crush injury	119 (0.2)
Amputation	34 (0.1)
**Operation**	
Yes	11,788 (21.3)
No	43,491 (78.7)
**Outcome**	
Survived	54,609 (98.8)
Died	670 (1.2)
**Primary site of injury**	
Head	11,050 (20.0)
Neck	10,523 (19.0)
Spine	7 (0.0)
Chest	4625 (8.4)
Abdomen/Back	11,129 (20.1)
Shoulder/Upper Arm	4125 (7.5)
Forearm	1660 (3.0)
Wrist/Hand	1669 (3.0)
Hip/Thigh	1580 (2.9)
Knee/Lower Leg	5756 (10.4)
Ankle/Foot	2136 (3.9)
Multiple Sites	933 (1.7)
Unknown site	86 (0.1)
**Role in accident**	
Driver	22,358 (40.4)
Pedestrian	9411 (17.0)
Public transit Passenger	8818 (16.0)
Car passenger	5795 (10.5)
Person injured while boarding or exiting vehicle	5699 (10.3)

In the comparisons of primary and additional diagnostic codes for severe and mild injuries, primary diagnoses for severe injuries were concentrated mostly in minority codes, such as “injuries to the hip and thigh” (S720, S723 and S724). Additional diagnoses for severe injuries were concentrated in “fracture of neck” (S122), “sprain of cervical spine” (S134), “fracture of shoulder” (S420), and “injury of muscle and tendon at hip and thigh” (S764). Contrastingly, the codes for “sprain of cervical spine” (S134) and “sprain of lumbar spine” (S335) appeared with high frequency as primary diagnoses for mild injuries, while codes for “contusion of knee” (S800), “sprain of cervical spine” (S134), and “hypertension” (I109) appeared with high frequency as additional diagnoses.

Of the individual primary diagnostic codes that summed up to 200 or higher in frequency, any same codes did not appear between mild and severe injuries. In addition, of the individual additional diagnostic codes that summed up to 20 or higher in frequency, there were no same codes between mild and severe injuries, except for S134 (“sprain and strain of cervical spine”). Thus, the results demonstrate differences in the distribution of codes for mild and severe injuries. When the additional diagnostic codes of these deceased patients were analyzed, a variety of them appeared, but the codes for diabetes mellitus, hypertension, and head injury appeared with high frequency.

**Fig. 2:**
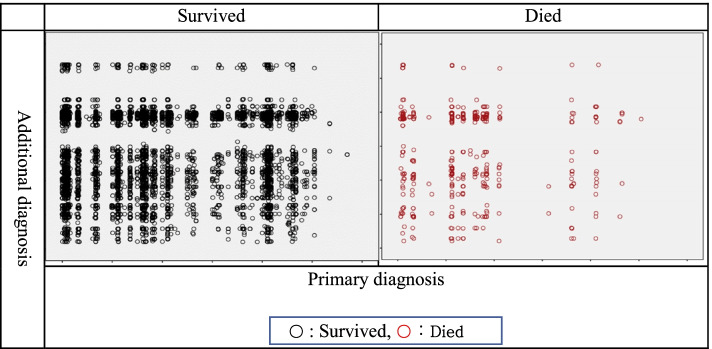
Mortality distribution

After classifying mortality as “survived” and “died,” scatter plots were drawn with primary diagnosis as the X axis and additional diagnosis as the Y axis (Fig. 2). The results showed that there were no deaths in the “Injury of shoulder and upper arm, elbow and forearm, wrist and hand” (S400 ~ S699) category of primary diagnosis according to mortality (1: survived, 2: died) in primary diagnoses. Moreover, there were no deaths in the “Infections and parasitic diseases” (A09 ~ A39, A490-A530, B009 ~ B09 and B181 ~ B86) and “Neoplasms” (C17 ~ D48) categories of the additional diagnoses. The results indicated a difference in the distribution of data in the two samples classified according to mortality.

Examination of differences according to the pattern of injury, notwithstanding the differences according to mortality, showed that “superficial injury,” “open wound,” and “sprain and dislocation,” which are classified as mild injuries, accounted for 63% of total 34,826 cases. Therefore, it was very rare for death to occur in patients with a mild injury and no underlying disease. After classifying the complete sample data into mild and severe injuries, cross analysis was performed with mortality status. Pearson’s chi-squared was 952.207, which was significant at a level of 0.01. This indicated that the frequency of mortality among patients with mild injury was statistically different when compared to that of severe injury. Cross analysis results showed that in the mild injury group (*n* = 34, 826), 39 patients (0.1%) died, while in the severe injury group 3.1% of the patients died. Accordingly, it was determined that there was a major difference in the rate of mortality corresponding to the severity of injury.

### Importance analysis

The present study used eight predictor variables including personal characteristics, injury caused by an accident, and disease factors to perform importance analyses. For the importance analyses, data over-sampled by SMOTE were used and analyses were performed with three different classification algorithms. To use the same variables in the comparison of performance between classification algorithms, variables below 10% of importance in all classification algorithms were excluded.

**Fig. 3:**
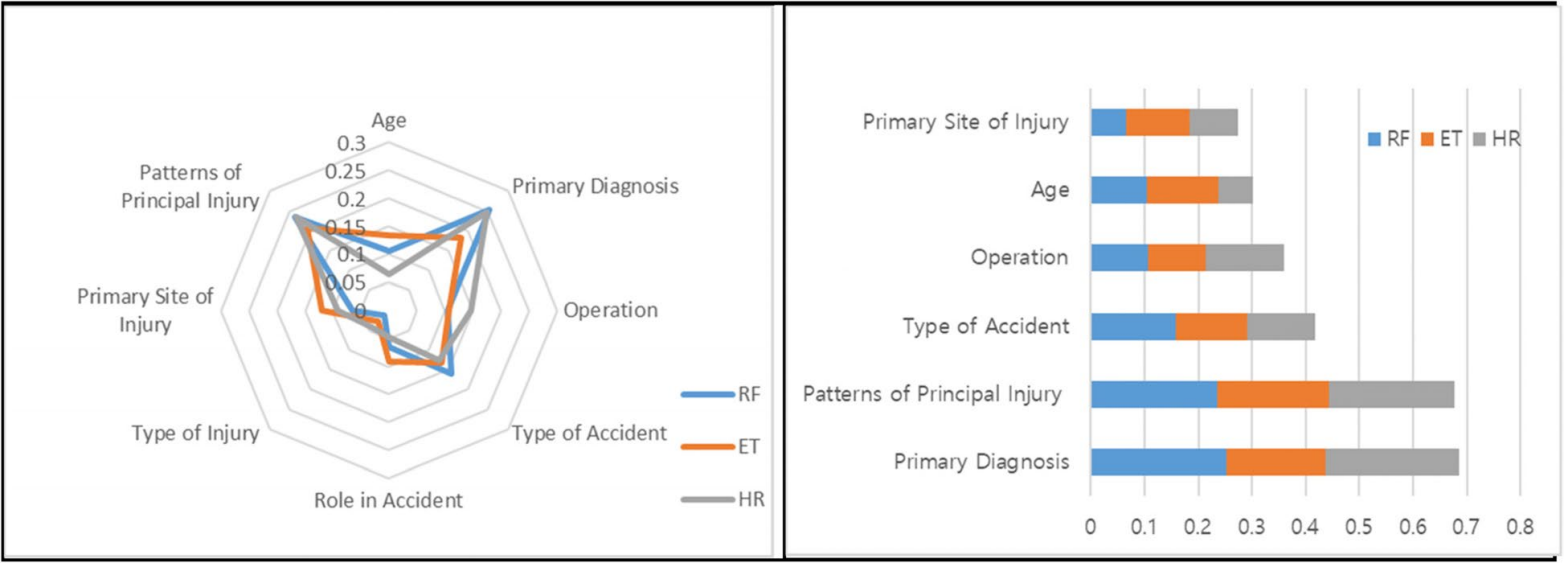
Importance analysis results

Based on the analysis results, six items, excluding type of injury and role in accident, were selected. Primary diagnosis and patterns of principal injury had the highest importance; type of accident and operation had moderate importance; and age and primary site of injury had relatively low importance. Moreover, there were differences in the importance according to the features of classification algorithms. For example, the pattern of the principal injury showed a high importance in all three algorithms, whereas primary diagnosis showed high importance in RF and XGBoost, but relatively low importance in Extra-Trees. Moreover, operation showed a higher importance with XGBoost than the other two algorithms, while age and primary site of injury showed a higher importance with Extra-Trees than the other algorithms (Fig. 3).

### Comparison of performance between ensemble algorithms

Among the ensemble techniques for classification used Random Forest, Extra-Trees, and XGBoost. Four different over- and under-sampling techniques were used to compare the performance of algorithms using accuracy, precision, recall, F1, and MCC.

**Fig. 4:**
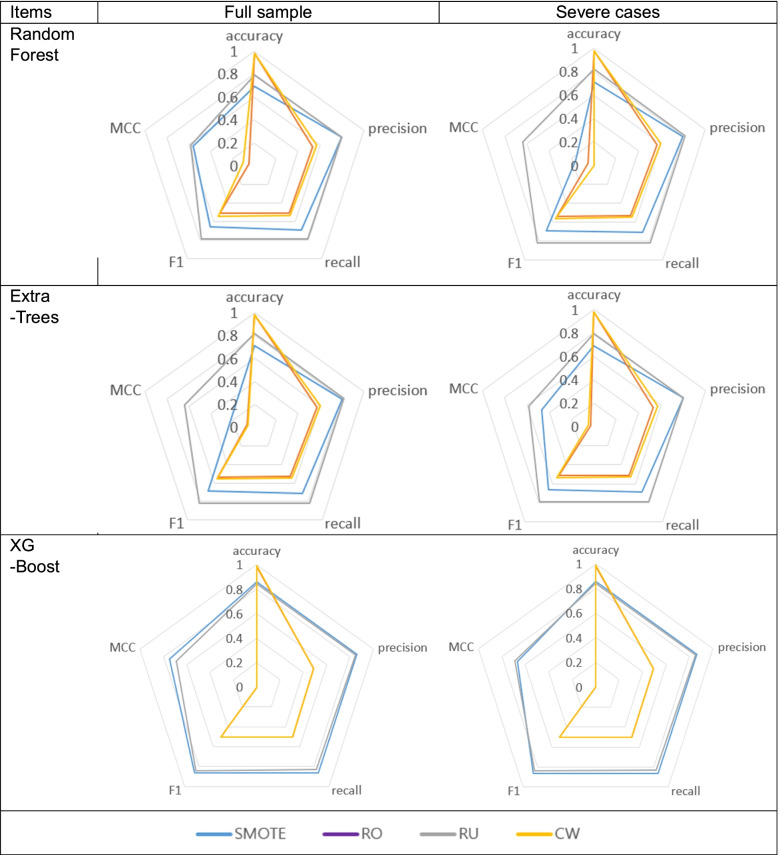
Comparison of algorithm performance

When the performance of three algorithms were compared (Fig. 4), the samples corrected using random-over and cost-sensitive learning showed the highest accuracy, but the assessment indicators using precision, recall and F1were lower than in SMOTE and random-under techniques. Moreover, SMOTE and random-under showed similar patterns in four assessment indicators. In the analyses using Random Forest and Extra-Trees algorithms, random-under technique showed superior performance than the SMOTE. However, in the analysis using the XGBoost algorithm, there was no significant difference between the SMOTE and random-under sampling techniques, but the XGBoost was found to show slightly superior performance in all four indicators, including accuracy. With respect to accuracy, which is a general performance assessment indicator, samples corrected using cost-weight technique showed excellent accuracy, and in particular, best accuracy of 99% was recorded when XGBoost was used. However, accuracy indicators have limitations in imbalanced samples, and thus, it is important to test the performance using other indicators such as precision, recall, F1, and MCC. With respect to these three performance indicators, the best performance of 86% was obtained with samples corrected using the SMOTE and analyzed using the XGBoost algorithm.

Based on patterns of principal injury, mortality prediction was measured in 20,263 patients that were classified as having “severe injury”, after excluding “mild injuries”. The results showed improvement in performance indicators, as compared to when the full model was used. In particular, improvement in performance indicators was achieved especially when samples were corrected using random-over sampling and cost-weight techniques.

### Analysis of differences between sample correction techniques

**Fig. 5:**
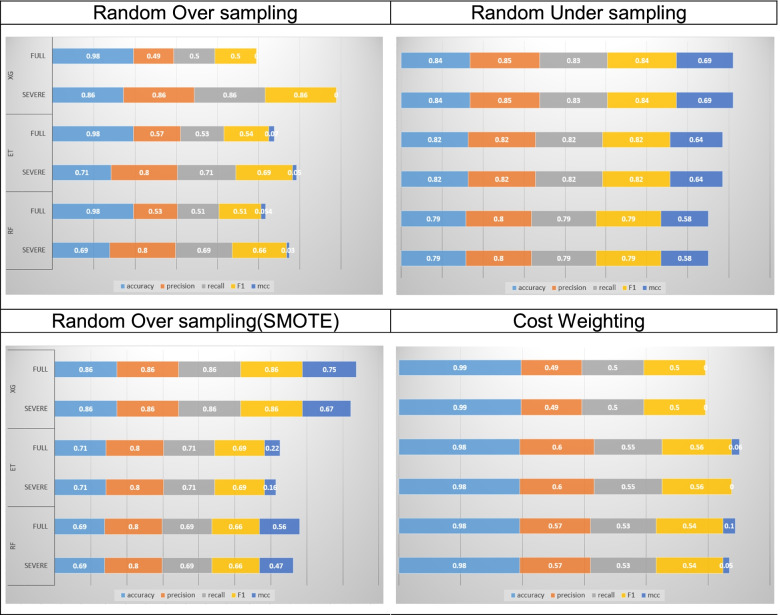
Comparison of sample correction methods

For sample correction, three over-sampling and one under-sampling techniques were used. Three over-sampling techniques used were random over-sampling, SMOTE, and cost-sensitive learning. The analysis results showed differences in the performance indicators in the assessment by sample correction technique (Fig. 5).

For the most general indicator “accuracy,” samples corrected using the cost-sensitive learning showed good accuracy; however, samples corrected using the random-under sampling technique showed superior results for the three performance assessment indicators other than accuracy.

In particular, the performance of the random-under sampling technique could be viewed as being excellent considering that “accuracy” indicator has limitations in imbalanced samples; “precision” and “recall” are important indicators; and “F1,” “MCC” are also a chief indicator. Therefore, for imbalanced data such as RTA mortality, under-sampling would be preferable over over-sampling.

When the full sample was used, accuracy was high, but “precision,” “recall,” and “F1” were low, which was viewed as a problem due to imbalanced sample. When analysis was performed with samples consisting of severe injuries, “accuracy” was high, but “precision,” “recall,” and “F1” values improved. Accordingly, the problem due to imbalanced sample was alleviated.

## Discussion

The present study implemented imbalanced sample correction and ensemble classification techniques to predict the performance of RTA mortality classification. The data used in the study spanned five years (2013–2017) and was extracted from KNHDS data collected by the Korea Disease Control and Prevention Agency. Among the KNHDS items, data regarding patient demographics, accident circumstances, and injury/disease characteristics were used to investigate the factors influencing mortality, which was the classification criterion.

There were 1030 primary diagnostic codes for 55,279 RTA trauma patients, which made up the sample in the present study. Of these, “intracranial injury” (S065, S066 and S062) showed a high frequency; 58.7%, patients were assigned the top 20 primary diagnostic codes, which were similar to external injuries caused by RTA. Moreover, among additional diagnoses, “hypertension” (I109), “contusion of knee” (S800), “multiple superficial injuries” (T009), “sprain of cervical spine” (S134), and “type II Diabetes Mellitus” (E119) showed high frequency; 875 codes with a frequency of one were included and the total number of additional diagnostic codes was 7673.

Among the primary diagnostic codes, “intracranial injury” (S061, S068) and “multiple fracture of ribs” (S224) showed high mortality rate, while “pleurisy” (J90) and “pneumonia” (J189) codes showed high mortality rate among the additional diagnostic codes. Such differences in mortality rates according to codes were confirmed through scatter plots using primary and additional diagnoses as the axes. Moreover, injuries that were classified as mild injuries, such as “superficial injury,” “open wound,” and “sprain and dislocation,” showed lower mortality rate than severe injuries. Moreover, the average age of patients with mild injury was 62.28 years, which was considerably higher than the average age of 42.79 years for the complete sample population. Among the patients who died, there were many who had underlying diseases, such as hypertension and diabetes mellitus. For external causes of injury, such as in RTA, with large differences in mortality rate according to diagnostic code and type of injury, we hypothesized that prediction of mortality by classifying the patients according to severity would produce more accurate results.

The RTA samples extracted from KNHDS were large in size, but the mortality rates were imbalanced. Moreover, considering previous studies reporting that distortion may occur during accuracy measurement when raw data are used for classification prediction of imbalanced data, imbalanced data were corrected using over- and under-sampling techniques. Furthermore, the variables used in the analysis were standardized considering that they have varying characteristics and units. Algorithms, such as Random Forest, Extra-Trees, and XGBoost, were also used considering the outstanding performance of ensemble algorithms in classification.

Comparison of the performance of the classification algorithms showed differences between performance assessment indicators according to the algorithm. Accuracy, which is the most general performance assessment indicator, was highest with all classification algorithms in samples corrected using random-over and cost-sensitive learning, which confirmed the significance of the model. However, other assessment indicators besides accuracy, meaning “precision,” “recall,” and “F1,” were low. Considering that previous studies have reported that using “precision,” “recall,” ““F1,” and “MCC” indicators are more valid than using “accuracy” for imbalanced data, it was determined that there are limitation in using “accuracy.”

Among the sample correction techniques, SMOTE and random-under did not show distortion that was as severe as the random-over and cost-sensitive learning and showed similar patterns in four model performance indicators. Among the two sampling techniques, random-under technique was superior than SMOTE in analyses using Random Forest and Extra-Trees algorithms. While there were no significant differences between the SMOTE and random-under techniques in the XGBoost algorithm, the XGBoost technique was slightly superior for all four indicators, including accuracy.

Accuracy results were excellent in samples that were sampled using cost-sensitive learning, and in particular, the best “accuracy” of 99% was recorded when XGBoost was used. However, considering that “precision,” “recall,” “F1,” and “MCC” are more important than “accuracy” in imbalanced samples, under-sampling was superior than over-sampling. However, the optimal combination between sampling technique and classification algorithm was samples corrected using the SMOTE technique and analyzed using the XGBoost algorithm.

To conclude, analysis of types of injuries caused by RTA, while excluding mild injuries classified as “superficial injury,” “open wound,” and “sprain and dislocation” that have low association with mortality, showed that “precision,” “recall,” and” “F1,” and “MCC” which are performance indicators other than “accuracy” in imbalanced samples improved in performance relative to the full sample. In all three algorithms used in the present study, performance improvement relative to the full sample and the XGBoost algorithm was found to be slightly superior.

These findings also highlight the need to classify management methods according to the types of injuries when managing RTA patients and to identify and control the RTA environment that leads to severe injuries. Moreover, from a statistical methodology perspective, there is also the need to conduct additional analyses using SVM, or other sampling techniques, and feature selection methods such as SHAP and LIME. In addition, since this research data is periodically collected national statistical data, the data of the next cycle can be used for verification.

The present study was significant as it presented the results of an empirical comparison of the validity of sampling techniques and classification algorithms that affect the accuracy of imbalanced samples by combining two techniques. These findings can be used as reference data in classification analyses of imbalanced data in the medical field.

## Data Availability

The datasets used and/or analyzed during the current study are available from the corresponding author on reasonable request.

## Abbreviations

RTA: Road traffic accidents
KHNDS: Korean National Hospital Discharge In-depth Injury Survey
SMOTE: the synthetic minority oversampling technique

## References

[1] Grossman MD, Reilly PM, Gillett T, Gillett D (1999). National Survey of the incidence of cervical spine injury and approach to cervical spine clearance in U.S. trauma centers. J Trauma.

[2] Davis JW, Phreaner DL, Hoyt DB, Mackersie RC (1993). The etiology of missed cervical spine injuries. J Trauma.

[3] Sanchez B, Waxman K, Jones T, Conner S, Chung R, Becerra S (2005). Cervical spine clearance in blunt trauma: evaluation of a computed tomography-based protocol. J Trauma.

[4] Rayan JA, Virginia L, Charne M (2022). A state-of-the-art review of factors that predict mortality among traumatic injury patients following a road traffic crash. Aust Emerg Care.

[5] Desai RJ, Wang SV, Vaduganathan M, Evers T, Schneeweiss S (2020). Comparison of machine learning methods with traditional models for use of administrative claims with electronic medical records to predict heart failure outcomes. JAMA Netw Open.

[6] Nistal-Nuño B (2022). Developing machine learning models for prediction of mortality in the medical intensive care unit. Comput Methods Programs Biomed.

[7] Wei C-P, Chiu I-T (2002). Turning telecommunications call details to churn prediction: a data mining approach. Expert Syst Appl.

[8] Coussement K, Van den Poel D (2008). Churn prediction in subscription services: an application of support vector machines while comparing two parameter-selection techniques. Expert Syst Appl.

[9] Mozer MC, Wolniewicz R, Grimes DB, Johnson E, Kaushansky H (2000). Predicting subscriber dissatisfaction and improving retention in the wireless telecommunications industry. IEEE Trans Neural Netw.

[10] Geurts P, Ernst D, Wehenkel L (2006). Extremely randomized trees. Mach Learn.

[11] Dhaliwal SS, Nahid AA, Abbas R (2018). Effective intrusion detection system using XGBoost. Information.

[12] Roshan SE, Asadi S (2020). Improvement of bagging performance for classification of imbalanced datasets using evolutionary multi-objective optimization. Eng Appl Artif Intell.

[13] Blagus R, Lusa L (2013). Improved shrunken centroid classifiers for high-dimensional class-imbalanced data. BMC Bioinform.

[14] Lopez V, Fernandez A, Garcia S, Palade V, Herrera F (2013). An insight into classification with imbalanced data: empirical results and current trends on using data intrinsic characteristics. Inf Sci.

[15] He H, Garcia V (2009). Learning from imbalanced data. IEEE TKDE.

[16] Blagus R, Lusa L (2013). SMOTE for high-dimensional class-imbalance data. BMC Bioinformatics.

[17] Garcia S, Herrera F (2009). Evolutionary under-sampling for classification with imbalanced data sets: proposals and taxonomy. Evol Comput.

[18] Bach M, Werner A, Zywiec J, Pluskiewicz W (2017). The study of under- and over-sampling methods’ utility in analysis of highly imbalanced data on osteoporosis. Inf Sci.

[19] Leonard KJ, Rauner MS, Schaffhauser-Linzatti MM, Yap R (2003). The effect of funding policy on day of week admissions and discharges in hospitals: the cases of Austria and Canada. Health Policy.

[20] Freitas A, Silva-Costa T, Lopes F, Garcia-Lema I, Teixeira-Pinto A, Brazdil P, Costa-Pereira A (2012). Factors influencing hospital high length of stay outliers. BMC Health Serv Res.

[21] Kim SS, Kim WJ, Kang SH (2011). A study on the variation of severity adjusted LOS on Injry inpatient in Korea. J Korea Acad Indust Coop Soc.

[22] Song YR, Lee MS, Kim DR, Kim KH (2017). A convergence study on the characteristics of length of hospita l stays of in jured and traumatic death patients-based on the Korea national hospital discharge injury survey data. J Korea Convergence Soc.

[23] M. Denil, T. Trappenberg. (2010) Overlap versus Imbalance. In: Farzindar A., Kešelj V. (eds) Advances in Artificial Intelligence. Canadian AI 2010. Lecture notes in computer science, vol 6085. Springer, Berlin, Heidelberg. 10.1007/978-3-642-13059-5_22.

[24] Beyan C, Fisher R (2015). Classifying imbalanced data sets using similarity based hierarchical decomposition. Pattern Recogn.

[25] Chawla NV, Bowyer KW, Hall LO, Kegelmeyer WP (2002). SMOTE: synthetic minority over-sampling technique. J Artif Intell Res.

[26] A. Liaw, M. Wiener. (2001) Classification and regression by RandomForest. Forest, 23. https://www.researchgate.net/publication/228451484_Classification_and_Regression_by_RandomForest

[27] Schapire RE (1990). "the strength of weak learnability" (PDF). Mach Learn.

[28] Chen Y (2014). Machine learning for large-scale genomics: algorithms, models and applications.

[29] Sree Divya K, Bhargavi P, and Jyothi S. XGBoost Classifier to Extract Asset Mapping Features, International Conference On Computational And Bio Engineering, 195–208.

